# Muscle Strength, Lipid Metabolism and Hepatic Steatosis Are Improved with Ursolic Acid Treatment in High-Fat Diet-Induced Obese Mice

**DOI:** 10.3390/nu17193158

**Published:** 2025-10-05

**Authors:** Dongyang Kang, Li Cao

**Affiliations:** 1 Sports Institute, Henan University of Science and Technology, Luoyang 471023, China; 9943199@haust.edu.cn; 2 College of Food and Bioengineering, Henan University of Science and Technology, Luoyang 471023, China

**Keywords:** ursolic acid, obesity, grip strength, lipid metabolism, hormone, mice

## Abstract

**Background/Objectives**: The prevalence of obesity globally has increased steadily in the past decades. Obesity, sarcopenic obesity (SO) and nonalcoholic fatty liver disease (NAFLD) commonly coexist. Ursolic acid (UA), a natural pentacyclic triterpenoid, has demonstrated potential anti-obesity properties. This study was designed to evaluate the anti-obesity efficacy of UA in a mouse model of high-fat diet (HFD)-induced obesity, with a particular focus on its impact on muscle function and NAFLD. **Methods**: Male C57BL/6J mice (6 weeks old) were randomly assigned to three groups (*n* = 20 per group): a control group (CON) fed a normal chow diet, a high-fat diet group (HFD), and a UA treatment group (UA). The HFD and UA groups received a high-fat diet for 10 weeks to induce obesity. Thereafter, mice in the UA group were administered UA orally once daily for 6 weeks. **Results**: In HFD-induced obese mice, UA administration significantly reduced body weight (BW), abdominal fat weight and liver weight; improved grip strength and muscle weight; and enhanced lipid profiles, including triglycerides, total cholesterol, low-density lipoprotein cholesterol and free fatty acid levels in serum. UA also improved histological changes in the liver and abdominal adipose tissues, regulated serum GH, IGF-1, T3, T4 and leptin levels and downregulated the inflammation-associated gene expression of TNF-α and IL-1β in abdominal adipose tissue. **Conclusions**: UA could enhance muscle strength, improve lipid metabolism and hepatic steatosis and might be considered a potential therapeutic agent for managing obesity and related metabolic diseases.

## 1. Introduction

Obesity is a major health problem worldwide. It is defined as abnormal or excessive fat accumulation with health risk by the World Health Organization (WHO) [1] and increases the risk of numerous diseases, including cardiovascular disease, type 2 diabetes, hyperlipidemia, fatty liver disease, cognitive impairments and various cancers [2,3,4]. The prevalence of obesity is dramatically increasing, and 20% of the world’s population will obese by 2030, according to projections [5]. Long-term over-nutrition, lack of exercise and an imbalance between energy intake and energy expenditure are factors that lead to obesity [6]. Obesity is also influenced by genetic factors [7]. Sarcopenia is defined as low muscle strength with the loss of skeletal muscle mass and function [8]. Meta-analyses have shown that sarcopenia and NAFLD have a positive association [9], and the cross-talk of cytokines produced by inflammatory reactions in skeletal muscle, liver and adipose tissue might form a triangle of harmful events that trigger the development and progression of obesity, sarcopenia and NAFLD [10]. Although sarcopenia is commonly associated with the elderly, recent research has shown that children may also develop the condition [11]. Sarcopenic obesity (SO), the coexistence of obesity and sarcopenia, is defined as obesity with low skeletal muscle mass and function [12]. Both obesity and sarcopenia synergistically exert an additional hazardous impact on metabolic disease or amplify the health risks [13,14]. In line with sarcopenia, SO is linked not only to the elderly population, but also the pediatric population and possess negative health outcomes in children and adolescents [15,16,17].

Skeletal muscle, constituting approximately 40% of total body mass, is a critical tissue responsible for a range of key physiological functions, including energy metabolism, thermogenesis and joint locomotion [18,19]. Skeletal muscle is also endocrine tissue and participates in body weight regulation by different signaling pathways [20,21,22]. Existing studies have shown a strong association between muscle strength, adipose tissue and nonalcoholic fatty liver disease [23]. The complex network relationships among them might be constructed by myokines, adipokines and hormones [24]. Skeletal muscle mass is decreased with weight loss, implying that loss of muscle mass and function should be minimized during the management of weight reduction. Grip strength is often used to measure muscular strength and muscle mass [25]. A recent study revealed a close correlation between grip strength and NAFLD risk [26]. Grip strength has been proposed as a tool for identifying metabolic syndrome and sarcopenia in the elderly [26,27]. Therefore, understanding the relationship between obesity and muscular strength is important.

To improve obesity, available treatment approaches for obesity or overweight include lifestyle corrections and the use of medications. Additionally, currently, limited scientific evidence, the lack of standardization and the paucity of clinical studies contribute to the challenges in formulating definitive WHO general recommendations for the use of natural products in this clinical field, which necessitates the design and development of natural products as potential anti-obesity agents for the therapeutic management of obesity [28,29,30].

Ursolic acid (UA), a natural pentacyclic triterpenoid, is widely present in a variety of medicinal plants, fruits and herbs [31], such as ginseng, pear apples, olives, plums, cranberries, rosemary, oregano and eucalyptus, calendula rosemarie [32,33]. So, UA is considered a component of the standard human diet. UA has gained significant attention in recent years due to its diverse biological functions and multiple pharmacological characteristics, such as anti-inflammation, anti-oxidation and anticancer effects [34,35,36]. Kunkel and colleagues highlighted that UA increases skeletal muscle mass in a mouse model of diet-induced obesity, identifying that the Akt molecular pathway is involved in muscle growth [37]. Sun and colleagues found that UA treatment significantly reduced the weight of the gastrocnemius muscle and lipid droplets in the same muscle by increasing the secretion of irisin and the expression of genes involved in adipocyte differentiation and lipogenesis [38]. Several studies have also shown that UA improved adipose tissue insulin resistance in aged rats and ameliorated HFD-induced hepatic steatosis and liver injury [39,40]. These results indicate that UA possesses anti-obesity and anti-inflammatory effects. However, UA’s mechanism of action has not been fully elucidated, and the effect of UA on muscle strength and NAFLD requires further verification during anti-obesity treatment. In addition, hormones play pivotal roles in maintaining skeletal muscle homeostasis, and further research is needed to confirm the regulatory effects of UA on hormones.

This study aimed to investigate anti-obesity effect of UA in HFD-induced obese mice with a specific dose for six weeks. In addition, this study aimed to examine the influence of UA on muscle performance and NAFLD. These data will provide evidence for the use of UA as a dietary supplement in preventing and treating obesity and its related metabolic syndrome.

## 2. Materials and Methods

### 2.1. Chemicals

Ursolic acid (UA, purity ≥ 90.0%) was obtained from Sigma-Aldrich (Shanghai, China) Trading Co., Ltd. The dose of UA (40 mg/kg body weight) was selected based on previous literature [41,42].

### 2.2. Animal Experiments

All animal experiments received approval from the Institutional Animal Care and Use Committee of Henan University of Science and Technology (Approval No: 202403017; Date: 8 March 2024) and were performed in accordance with the Guide for the Care and Use of Laboratory Animals: Eighth Edition [43].

### 2.3. Study Design

Male specific pathogen free C57BL/6J (6 weeks old) mice were selected from Si Pei Fu (Beijing) Biotechnology Co., Ltd. (Beijing, China). All animals were housed in cages, and they were acclimated to the environment with automatic temperature, light and humidity control for 1 week. The room temperature was maintained at 24 °C and the humidity was controlled at 55%, with a 12-h light/dark cycle.

**Figure 1:**
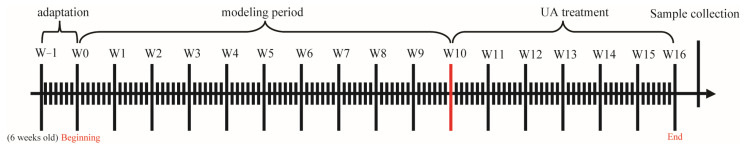
The time schedule of the procedures in this study.

A total of 60 mice were randomly assigned to three experimental groups (*n* = 20 per group): (1) control (CON), (2) high-fat diet (HFD) and (3) UA treatment (UA). The CON group received a standard chow diet (LAD3001M, Trophic Animal Feed High-Tech Co., Ltd., Nantong, China), with a nutritional composition of 67% carbohydrate, 18% protein and 5% fat, providing 7% of calories from fat. The HFD and UA groups were fed a high-fat diet (TP23400, Trophic Animal Feed High-Tech Co., Ltd, Nanning, China.) containing 23% protein, 32% carbohydrate, 31.5% fat (27.5% lard + 4.0% soybean oil), 6.8% fiber, 1.5% choline chloride, 4.0% mineral mix and 1.2% vitamin mix, with 60% of calories derived from fat. The mice were housed with five replicate cages per group (four mice per cage) and were allowed ad libitum access to food and tap water. After 10 weeks, the UA group was administered ursolic acid (40 mg/kg body weight) once daily via oral gavage, whereas the CON and HFD groups received an equivalent volume of saline for a duration of 6 weeks. Body weight and food intake were recorded weekly. The experimental timeline is summarized in Figure 1.

### 2.4. Grip Strength Test

Prior to the endpoint of this study, forelimb grip strength was evaluated in all mice using a specialized grip strength meter (RWD Life Science Co., Ltd., Shenzhen, China). During testing, each mouse was allowed to grasp a metal grip with both forepaws, after which the tail was gently pulled backward horizontally until the animal released its grip. The maximum force exerted immediately before release was recorded. Each mouse underwent three consecutive trials separated by one-minute intervals, and the average of these measurements was calculated to represent grip strength. 

### 2.5. Sample Collections

At the end of the experiment (after UA treatment for 6 weeks), the mice were anaesthetized with pentobarbital sodium after 12 h of overnight fasting. Blood from the hearts was collected using vacutainer tubes, and serum samples were obtained from the collected blood samples through 10 min of centrifugation (3500× *g*, 4 °C). Then, the triceps, gastrocnemius, quadriceps and soleus muscles, abdominal adipose tissues and liver tissues were removed, weighed and processed for subsequent analyses. In addition, the liver index was calculated as follows:

Liver index = liver weight/body weight

### 2.6. Measurement of Serum Lipids

The levels of total cholesterol (TC), triglyceride (TG), high-density lipoprotein cholesterol (HDL-C), low-density lipoprotein cholesterol (LDL-C) and free fatty acid (FFA) in serum were determined using commercial assay ELISA kits, according to the manufacturer’s instructions (Nanjing Jiancheng Bioengineering Institute, Nanjing, Jiangsu, China).

### 2.7. Observation and Analysis of Histopathology

Liver and abdominal subcutaneous adipose tissue samples were collected and fixed in 10% neutral buffered formalin. After 48 h of fixation, the tissues were dehydrated, embedded in paraffin and sectioned at a thickness of 5 µm. Following deparaffinization in xylene and rehydration, the sections were stained with hematoxylin and eosin (H&E). Liver histopathology was examined using light microscopy, with hepatic steatosis defined as the presence of lipid droplets in 5% or more of hepatocytes [44]. In brief, steatosis was assayed in three zones of the classic lobules of liver in each field: zone I (port area), zone II (intermediate area) and zone III (central vein area). When the total steatosis area in the three zones was ≥ 5%, it was recorded. For adipose tissue, images of the slides were captured, twenty cells were randomly selected from each slide and the diameter was measured. ImageJ software (1.49v) was used for the analysis of steatosis area and the diameters of adipocytes.

### 2.8. Measurement of Serum Hormones

Serum levels of thyroxine (TH, T3, T4), leptin, growth hormone (GH) and insulin-like growth factor-1 (IGF-1) were measured using corresponding commercial ELISA kits from Nanjing Jiancheng Bioengineering Institute.

### 2.9. Real-Time PCR

Total RNA was extracted from abdominal adipose tissue using TRIzol reagent (TaKaRa Bio Inc, Otsu City, Japan), following the manufacturer’s instructions. Subsequent cDNA synthesis was carried out with oligo-dT primers and PrimeScript RT enzyme (TaKaRa). Quantitative real-time PCR was performed on a Bio-Rad iQ5 detection system using SYBR Premix ExTaq II (TaKaRa) to evaluate the expression of inflammatory cytokines. All reactions were conducted in triplicate and normalized to the endogenous control, β-actin. The primer sequences utilized are listed in Table 1, and relative gene expression levels were calculated using the 2^−ΔΔCt^ method [45].

### 2.10. Statistical Analysis

All data were assessed for normality using the Kolmogorov–Smirnov test and are presented as mean ± standard deviation (SD). Statistical comparisons were performed using one-way ANOVA with Tukey’s post hoc test for multiple comparisons, using SPSS 22.0 software. Differences were considered statistically significant at *p* < 0.05.

## 3. Results

### 3.1. The Effect of Ursolic Acid on Body Weight and Feed Intake

**Figure 2:**
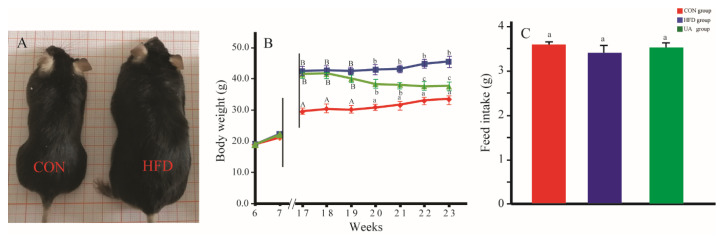
Changes in body weight and feed intake. (**A**): Representative images of C57BL/6J mice fed a standard chow diet and a high-fat diet for 10 weeks. (**B**): Body weight changes during the 6 weeks of UA administration. (**C**): Food intake during the 6 weeks of UA administration. Data are expressed as mean ± SD. Values with different capital letters are significant at the 0.01 level. Values with different lowercase letters are significant at the 0.05 level.

At the beginning of the experiment (week 0), no significant differences in body weight were observed among the different groups (*p* > 0.05). However, following 10 weeks of dietary intervention, mice fed a high-fat diet exhibited a significant increase in body weight compared to those maintained on a standard chow diet (Figure 2A). After treatment with UA for 6 weeks (Week 16), mice in the HFD group had higher body weight than mice in the CON group. However, the body weight of mice in the UA group was lower than that of mice in the HFD group (Figure 2B). No significant differences for feed intake were observed among the three experiment groups (Figure 2C).

### 3.2. The Effect of Ursolic Acid on Abdominal Fat and Liver Weight

**Figure 3:**
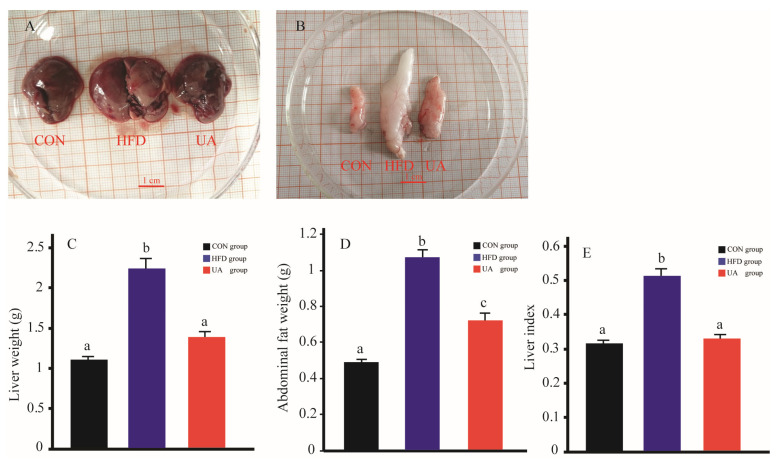
Changes in abdominal fat and liver weight. (**A**): Representative gross images of liver. (**B**): Representative gross images of abdominal subcutaneous adipose tissue. (**C**): Liver weight. (**D**): Abdominal fat weight. (**E**): Liver index. Data are expressed as mean ± SD. Different letters represent significant differences at the 0.05 level.

At the end of the experiment, the abdominal subcutaneous adipose tissue weight, liver weight and live index value of the mice in the HFD group were higher liver than those in the CON group. In contrast, the mice administered 40 mg/kg/day UA had lower liver weight, abdominal fat weight and liver index values than those in the HFD group (Figure 3C–E).

### 3.3. The Effect of Ursolic Acid on Grip Strength and Muscle Weight

At the end of the experiment, grip strength and muscle weights were measured in this study. The grip strength of mice in the HFD group was significantly lower than that in the CON group, and UA administration led to a significant increase compared with the HFD group (*p* < 0.05). Lower weights of the triceps, quadriceps and gastrocnemius, but not soleus, were observed in the HFD group compared with the CON group. UA administration significantly increased the weights of these muscles compared with those in the HFD group. These results are presented in Table 2.

### 3.4. The Effect of Ursolic Acid on Serum Lipids

We analyzed serum TG, TC, HDL-C, LDL-C and FFA levels. As shown in Table 3, these parameters in the HFD group showed significant increases compared with those in the CON group (*p* < 0.05). Treatment with UA significantly decreased TG, TC, LDL-C and FFA levels (*p* < 0.05), but not HDL-C.

### 3.5. The Effect of Ursolic Acid on Histological Changes in Liver and Abdominal Adipose Tissues

**Figure 4:**
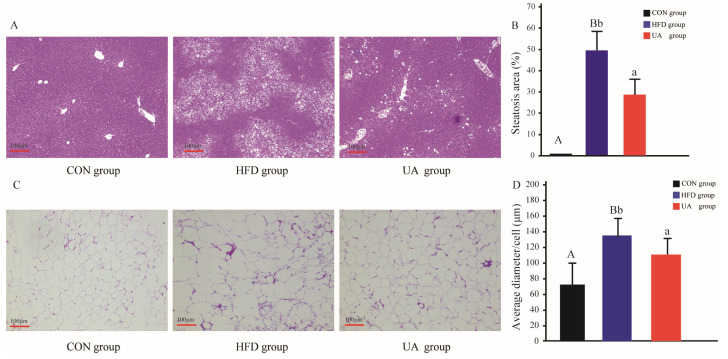
Histopathological changes in liver and abdominal adipose tissues. (**A**): Representative liver histology by H&E staining. (**B**): Steatosis area of the liver. (**C**): Representative adipose tissues.(**D**): Average diameter of adipocytes. Values with different capital letters are significant at the 0.01 level. Values with different lowercase letters are significant at the 0.05 level.

Histopathological assessment of liver tissues indicated that mice in the HFD group developed severe hepatic steatosis, exhibiting significantly larger steatotic areas (*p* < 0.01), along with substantial lipid droplet accumulation and hepatocellular swelling relative to the CON group (Figure 4A). In contrast, UA treatment effectively ameliorated these pathological changes, resulting in a noticeable reduction in both the number and size of intra-cellular lipid vacuoles. The steatotic area was markedly decreased from 53% in the HFD group to 22% in the UA-treated group (Figure 4B).

Morphometric analysis of abdominal adipose tissue further revealed adipocyte hypertrophy in the HFD group compared with the CON group. UA intervention significantly attenuated this increase in adipocyte size, as demonstrated by a clear reduction in the cross-sectional area of adipocytes in the UA group relative to the HFD group (Figure 4C,D).

### 3.6. The Effect of Ursolic Acid on Serum Hormones

**Figure 5:**
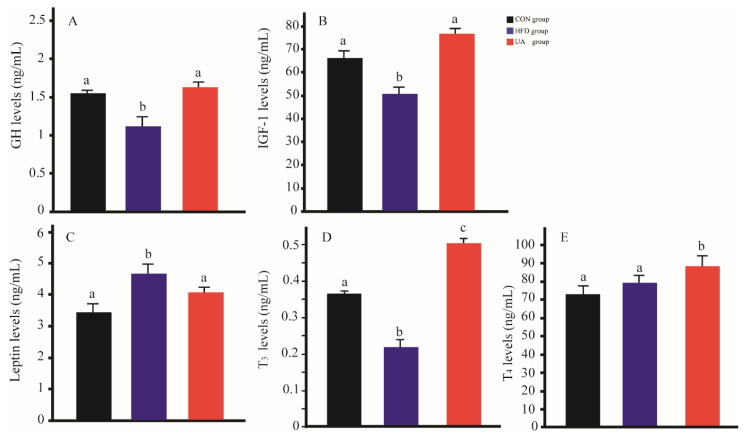
The effect of ursolic acid on serum hormone levels. (**A**) Serum GH levels. (**B**): Serum IGF-1 levels. (**C**): Serum leptin levels. (**D**): Serum T3 levels. (**E**): Serum T4 levels. Different letters represent significant differences at the 0.05 level.

To investigate the potential involvement of endocrine factors in obesity development, serum levels of leptin, T3, T4, GH and IGF-1 were measured. As shown in Figure 5, the HFD group exhibited notably reduced concentrations of GH, IGF-1 and T3 compared with the CON group (*p* < 0.05). In contrast, leptin levels were significantly elevated in HFD-fed mice relative to the control group (*p* < 0.05). After UA treatment, GH, IGF-1 and T3 levels were increased, and leptin levels were decreased (*p* < 0.05). Additionally, no significant difference was observed for T4 levels between the CON group and he HFD group, but the UA group showed a notable increase.

### 3.7. The Effect of Ursolic Acid on the Expression of Inflammation-Associated Genes in Abdominal Adipose Tissue

**Figure 6:**
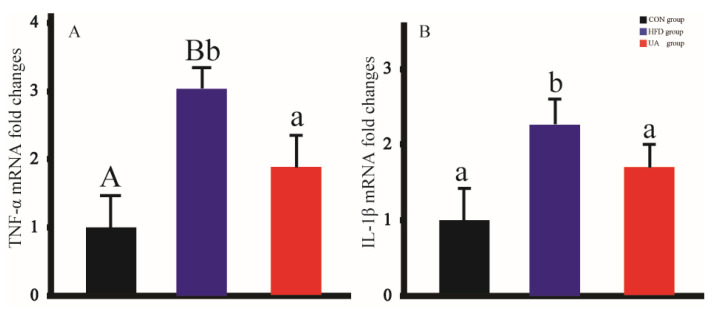
Changes in the expression of inflammation-associated genes. (**A**): TNF-α mRNA. (**B**): IL-1β mRNA. Values with different capital letters are significant at the 0.01 level. Values with different lowercase letters are significant at the 0.05 level.

We analyzed the expression of inflammation-associated genes TNF-α and IL-1β. The expression of TNF-α and IL-1β was significantly higher in the HFD group than in the CON group. UA administration significantly reduced their mRNA expression compared with the HFD group. The results are presented in Figure 6.

## 4. Discussion

Obesity represents a significant public health concern affecting elderly populations, children, and adolescents globally. Although body weight reduction is beneficial for managing obesity and mitigating associated metabolic disorders, it is frequently accompanied by a decline in skeletal muscle mass [46]. Skeletal muscle mass is closely related to function and metabolic capacity of the muscle [47], and skeletal muscle atrophy and dysfunction can further impair whole-body metabolism [48]. The desired outcomes for the management of people living with obesity are the maintenance or increase of muscle mass and function when body weight (fat mass) is reduced. Hand grip strength is a well-established indicator of muscle strength and is often used to assess muscle condition [49,50]. In the present study, the assessment of grip strength revealed a significant reduction in the HFD group. Notably, UA supplementation effectively restored grip strength and concurrently increased muscle mass in HFD-induced obese mice. Results regarding muscle strength and muscle mass are in line with human clinical studies, in which muscle strength was positively correlated with muscle mass [51,52,53].

Obesity is defined as a condition of excessive adipose tissue accumulation, characterized by adipose tissue expansion through adipocyte hypertrophy (increase in cell size) and/or hyperplasia (increase in cell number) [54,55]. The current study found that mice fed a HFD gained more body weight, abdominal fat mass and higher liver index values than those who received a control diet, and UA supplementation reduced body weight, abdominal fat mass and liver index values in HFD-fed mice. We also found that the adipocyte size in abdominal adipose tissue was decreased by UA, indicating that adipose tissue loss may have led to weight loss and that UA may regulate lipid metabolism in peripheral adipose tissues. The lipid abnormalities usually observed in obese patients are high levels of serum TG, TC, FFA and LDL-C [56,57]. Serum lipids were tested in the current study and our results demonstrated that UA effectively decreased TG, TC, LDL-C and FFA in serum in HFD-fed mice after 6 weeks of administration. This suggests that UA might be useful for treating and preventing hyperlipidemia.

Given the role of leptin and TH in the regulation of energy expenditure and lipid homeostasis, circulating leptin and TH levels were determined. Leptin is produced in adipose tissue and can regulate energy balance by inhibiting hunger and increasing energy expenditure in the body [58,59]. However, leptin levels seem to be increased in obese patients [60]. We also found that serum leptin levels are higher in HDF-fed mice. It is possible that high leptin levels lead to leptin resistance and blunt its positive effects [61,62]. TH was secreted by the thyroid gland. T3 is the most metabolically active form of TH, and T4 is considered a prohormone and constitutes a large reserve of T3. The regulation of energy expenditure is a critical role of TH [63]. Shao et al. reported that high-fat diets could induce hypothyroidism in mice by increasing thyrotropin and decreasing T3 and T4 levels, likely related to increased leptin signaling [64]. Consistent Shao et al.’s research, we found that T3 levels were lower in HDF-fed mice. UA treatment increased T3 and T4 levels. These data indicated that UA played a key role in energy homeostasis by regulating circulating hormone levels.

Both GH and IGF-1 are anabolic hormones related to skeletal muscle function and mass. Early and recent studies have shown that GH strongly affected skeletal muscle mass and performance [65,66]. GH can stimulate skeletal muscle protein synthesis and skeletal muscle growth in human and animals [67,68]. IGF-1 primarily regulates muscle mass through both the activation of protein synthesis and the inhibition of protein breakdown [69], and IGF-1 mediates the primary anabolic action of GH in skeletal muscle [70]. The reduction of GH/IGF-1 is markedly associated with a decrease in muscle growth and function in people with obesity [71]. In the present study, UA administration significantly elevated serum GH and IGF-1 levels compared with the HFD group, suggesting a plausible role of UA in enhancing muscle mass and function. Further investigation is warranted to elucidate the underlying mechanisms involved.

Obesity is a chronic metabolic disease accompanying low-grade inflammatory responses [72]. Excessive fat leads to the expansion of adipose tissue. Under these conditions, macrophage infiltration is triggered and levels of inflammatory cytokines (e.g., TNF-α and IL-1β) produced by activated macrophages are increased [73]. It has been demonstrated that TNF-α and IL-1β are two important pro-inflammatory biomarkers involved in the development of obesity [74,75,76]. Previous research has shown that TNF-α possesses higher expression levels in adipose tissue in obese and diabetic rodents [77], and increased TNF-α production in human adipose tissue is positively correlated with obesity [78]. IL-1β participates in the inflammatory process of obesity and obesity-related diseases [79] and contributes to the development of insulin resistance [80]. Our findings were consistent with previous research. Here, the HFD group showed a higher expression of TNF-α and IL-1β mRNA, whereas UA treatment down-regulated their mRNA levels. This demonstrates that UA possesses anti-inflammatory effects.

Nonalcoholic fatty liver disease (NAFLD) is closely associated with obesity [81]. When adipose tissue storage capacity becomes limited, ectopic fat deposition occurs in organs such as the liver, which may contribute to the onset and progression of NAFLD [82]. Duan et al. showed that HFD induced rapid weight gain and obesity and expanded areas of liver steatosis in mice [83]. Similarly to their study, the current study indicated that the mice fed a HFD had higher body weight and liver weight and bigger areas of liver steatosis. To test the ability of UA to limit liver steatosis, we evaluated the histological appearance of this organ in mice. The results showed that UA reduced liver weight and improved the histological appearance of the liver, indicating that UA reduced liver steatosis.

This study has potential limitations. Muscle strength was assessed at only a single terminal point; data from both baseline and the end of the experiment should be included. Although a dose of 40 mg/kg body weight is effective under the conditions of this study, this cannot be directly translated to dietary intake in humans; more clinical studies are needed in the future. In addition, doses of UA should be tested in order to establish dose–response effects. Energy expenditure could not be tested due to limitations of the experimental conditions, which limits further understanding of the management of obesity with UA treatment. The mass and functions of brown adipose tissues were not evaluated in this study, so further examination of brown adipose tissue might provide a stronger scientific basis for the application of UA. In addition, the mechanism of action of UA on different target organs also needs to be clarified.

## 5. Conclusions

In this study, we showed that UA enhanced grip strength and improved body weight and hepatic steatosis by altering muscle mass, fat mass, hormone and inflammatory cytokines in HFD-induced obese mice, demonstrating its efficacy against obesity and improving muscle function and the metabolic environment.

These findings confirmed that UA administration enhanced muscle strength and improved lipid metabolism and hepatic steatosis induced by HFD. These data also show that UA acts on multiple target organs and could be considered a potential therapeutic agent for managing obesity, sarcopenia and other metabolic diseases, or might serve as a functional ingredient in the development of health-promoting foods. Based on these findings, future studies are required to understand the underlying mechanisms.

## Figures and Tables

**Table 1:** Primer sequences for quantitative real-time PCR.

Gene	Forward Primer (5′−3′)	Reverse Primer (5′−3′)
IL-1β	TGCCACCTTTTGACAGTGATG	TTCTTGTGACCCTGAGCGAC
TNF-α	TCTTTCGGAGAAGGTTGCCC	AGAAGTCCTGCCACTTCACG
β-actin	AGAGGGAAATCGTGCGTGAC	CAATAGTGATGACCTGGCCGT

IL-1β: Interleukin-1β. TNF-α: Tumor necrosis factor-α.

**Table 2:** The effect of ursolic acid on grip strength and various muscle weights.

	Groups		
	CON	HFD	UA
Grip strength (g force (gf))	118.34 ± 13.24 ^a^	99.68 ± 14.71 ^b^	112.86 ± 11.52 ^a^
Triceps (mg)	234.77 ± 25.14 ^a^	198.64 ± 17.75 ^b^	242.54 ± 15.68 ^a^
Quadriceps (mg)	415.31 ± 39.03 ^a^	369.61 ± 38.73 ^b^	364.00 ± 27.38 ^b^
Gastrocnemius (mg)	288.61 ± 21.89 ^a^	271.64 ± 17.43 ^b^	290.29 ± 21.82 ^a^
Soleus (mg)	14.26 ± 1.06	16.58 ± 0.95	15.14 ± 1.32

Data are expressed as mean ± SD. Different letters represent significant differences at the 0.05 level.

**Table 3:** The effect of ursolic acid on plasma lipids.

	Groups		
	CON	HFD	UA
TG (mmol/L)	0.64 ± 0.04 ^a^	1.18 ± 0.07 ^b^	0.86 ± 0.05 ^a^
TC (mmol/L)	2.53 ± 0.28 ^a^	4.69 ± 0.23 ^b^	3.07 ± 0.31 ^a^
HDL-C (mmol/L)	2.34 ± 0.14 ^a^	3.64 ± 0.17 ^b^	3.24 ± 0.25 ^b^
LDL-C (mmol/L)	0.21 ± 0.03 ^a^	0.42 ± 0.05 ^b^	0.26 ± 0.08 ^a^
FFA (mmol/L)	0.37 ± 0.04 ^a^	0.78 ± 0.11 ^b^	0.44 ± 0.06 ^a^

Data are expressed as mean ± SD. Different letters represent significant differences at the 0.05 level.

## Data Availability

The original contributions presented in this study are included in the article. Further inquiries can be directed to the corresponding authors.

## Abbreviations

The following abbreviations are used in this manuscript:

SO	Sarcopenic obesity
NAFLD	Nonalcoholic fatty liver disease
UA	Ursolic acid
HFD	High-fat diet
TC	Total cholesterol
TG	Triglyceride
HDL-C	High-density lipoprotein cholesterol
LDL-C	Low-density lipoprotein cholesterol
TH	Thyroxine
GH	Growth hormone
IGF-1	Insulin-like growth factor-1
IL-1β	Interleukin-1β
TNF-α	Tumor necrosis factor-α
FFA	Free fatty acid
